# Hyperthermia Enhances Doxorubicin Therapeutic Efficacy against A375 and MNT-1 Melanoma Cells

**DOI:** 10.3390/ijms23010035

**Published:** 2021-12-21

**Authors:** Diana Salvador, Verónica Bastos, Helena Oliveira

**Affiliations:** Department of Biology and CESAM, University of Aveiro, 3810-193 Aveiro, Portugal; diana.s@ua.pt (D.S.); veronicabastos@ua.pt (V.B.)

**Keywords:** skin cancer, melanoma, hyperthermia, doxorubicin, cytotoxicity, cell cycle arrest, reactive oxygen species, apoptosis

## Abstract

Melanoma is the deadliest form of skin cancer, and its incidence has alarmingly increased in the last few decades, creating a need for novel treatment approaches. Thus, we evaluated the combinatorial effect of doxorubicin (DOX) and hyperthermia on A375 and MNT-1 human melanoma cell lines. Cells were treated with DOX for 24, 48, and 72 h and their viabilities were assessed. The effect of DOX IC10 and IC20 (combined at 43 °C for 30, 60, and 120 min) on cell viability was further analyzed. Interference on cell cycle dynamics, reactive oxygen species (ROS) production, and apoptosis upon treatment (with 30 min at 43 °C and DOX at the IC20 for 48 h) were analyzed by flow cytometry. Combined treatment significantly decreased cell viability, but not in all tested conditions, suggesting that the effect depends on the drug concentration and heat treatment duration. Combined treatment also mediated a G2/M phase arrest in both cell lines, as well as increasing ROS levels. Additionally, it induced early apoptosis in MNT-1 cells, while in A375 cells this effect was similar to the one caused by hyperthermia alone. These findings demonstrate that hyperthermia enhances DOX effect through cell cycle arrest, oxidative stress, and apoptotic cell death.

## 1. Introduction

Cancer is currently a severe health problem, being one of the main causes of death worldwide [1]. It defines a group of diseases characterized by the undisciplined growth and proliferation of abnormal cells, occurring in a variety of tissues and organs [2]. Skin cancer represents one of the most predominant forms of human cancer and one of the most expensive, and its incidence is increasing in the whole world due to ozone layer deterioration and consequent higher sun exposure [3]. Among skin cancers, malignant melanoma is one of the most threatening types due to its metastatic capacity, meaning that its prevalence is increasing at an alarming rate in Caucasians [4]. Melanoma is a malignant tumor that arises from melanocytes and can appear in both cutaneous and mucosal surfaces [5]. However, despite the higher mortality rates associated with mucosal melanoma, over 95% of melanomas are cutaneous [6,7]. Surgery is the usual approach for early-stage melanoma but its efficacy at advanced stages is low. Some non-surgical therapies may also be applied, such as radiotherapy, immunotherapy, photodynamic therapy, cryotherapy, and chemotherapy [8]. Notwithstanding, melanoma is one of the most difficult cancers to address therapeutically [9].

Doxorubicin (DOX) is a broad-spectrum anthracycline antibiotic obtained from the actinobacteria *Streptomyces peucetius* var. *caesius* [10,11]. Its principal target is DNA damage and the inhibition of macromolecular biosynthesis by the intercalation of DNA, and the inhibition of topoisomerase II, preventing replication [12,13]. After entering the cell, DOX binds to the proteasome with high affinity. Then, this complex translocates into the nucleus, where DOX dissociates and binds to DNA, obstructing the cleaving activity of topoisomerase II and disrupting nucleic acid synthesis [14,15]. Topoisomerase-II-mediated DNA damage leads to cell cycle arrest in G0/G1 and G2/M phases and programmed cell death [16]. In addition, anthracyclines can also bind to mitochondrial DNA (mtDNA) [17].

Currently, DOX is one of the most used agents for cancer treatment, including blood, breast, lung, ovarian, and bladder cancers [18,19,20]. However, the typical use of a single chemotherapeutic agent has some limitations, such as drug resistance, no lasting efficacy, and undesirable toxicity [21]. In fact, DOX induces severe side effects, such as medina fatal cardiomyopathy, if the lifetime cumulative total soluble dose transcends 450 mg/m^2^ [22,23,24], which is caused by the increased levels of free radicals and decreased levels of antioxidants, resulting in oxidative stress [25,26]. 

Therapeutic hyperthermia, defined as applying heat to treat a disease, is currently under considerable interest because of its improvements of the effects of chemotherapy in many tumors [27,28]. This enhanced response has been associated with the well-known biological effects of heat stress, namely the suppression of DNA damage repair via protein denaturation or inactivation; elevated drug delivery due to heat-induced increases in blood flow and vascular permeability; and the increased cellular uptake and increment of the direct cytotoxicity of the drug [29,30,31,32]. In fact, it has been proven that many drugs are potentiated by hyperthermia and that it aids in overcoming drug resistance [28]. Resistance is also a major issue in DOX therapy [33]. Some studies have demonstrated that DOX is less effective in melanoma treatment due to the high natural resistance of these tumor cells [34,35,36]. Usually, this resistance is related to transport proteins using DOX as a substrate, the activity of which decreases the drug’s accumulation in cells and reduces its cytotoxic effects [10,37,38]. A study with colorectal cancer has demonstrated that hyperthermia increases intracellular DOX concentrations [28]. In breast cancer, hyperthermia increased intracellular ROS production and downregulated ATP-binding cassette sub-family G member 2 (ABCG2) expression—an exporter of DOX—leading to cell damage enhancement via DOX [39]. In addition, Blasiak and colleagues demonstrated that DOX-resistant cancer cells exposed first to DOX and then to hyperthermia, needed double the amount of time to completely repair the DNA damaged by DOX, in comparison to their DOX-sensitive counterparts and to normal cells [40]. Hence, hyperthermia combined with DOX can improve responses to treatment and diminish side effects in normal cells. Thus, hyperthermia can theoretically help overcome the problems related to limited DOX dosage and resistance by improving the amount of drug that reaches and enters the cells. Therefore, we aimed to investigate the potentiated effect of low concentrations of DOX by hyperthermia in melanoma and assessed the potential molecular mechanisms behind the response.

## 2. Results

### 2.1. DOX Decreases A375 and MNT-1 Viability

**Figure 1:**
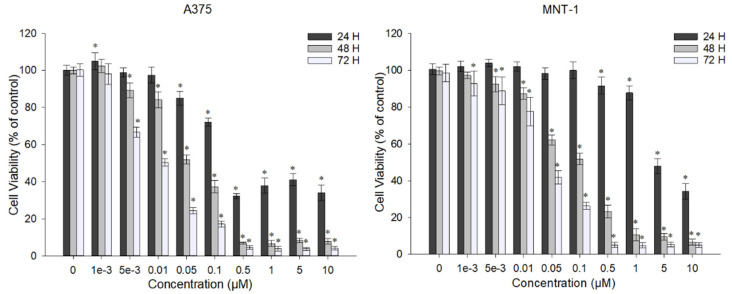
Effect of DOX on cell viability of A375 and MNT-1 cells. Cells were exposed to different concentrations of DOX for 24, 48, and 72 h, and cell viability was determined using MTT assay. Data shown are mean values ± standard deviation of three independent experiments with four technical replicates each. *—indicates statistical significance in comparison to the respective control (*p* < 0.05).

The chemotherapeutic agent DOX is used in the treatment of different types of cancer. To determine the effect of DOX in A375 and MNT-1 cells, cell viability was evaluated after treatment for 24, 48, and 72 h. As shown in Figure 1, in the A375 cell line, the lowest concentration caused a significant reduction effect after 24 h of exposure to 0.05 μM, reducing the cell viability to approximately 86%. Furthermore, the lowest concentration tested (0.001 μM) did not affected the cell viability even after 72 h of exposure. The MNT-1 cell line showed less sensibility to DOX, having a significant reduction (~8%) with exposure to the concentration of 0.5 μM for 24 h, compared to the control, which is 10-times higher than the concentration that caused a reduction in the case of A375. However, the lower concentration was able to significantly reduce cell viability after 72 h of exposure. The higher concentrations (≥1 μM) almost completely eliminated the cells after 48 h of exposure for both cell lines.

Table 1 shows that the ICs decreased when the exposure time increased. In addition, MNT-1 cells were less responsive to DOX, presenting higher ICs than A375 cells. In fact, the calculated IC_50_ for 24 h of exposure was 4.88 μM for MNT-1 cells and 0.45 μM for A375 cells, corresponding to a DOX IC almost 11-times higher for MNT-1 than for A375. Even in the longer exposure time, MNT-1 cells had a DOX IC_50_ 3.5-fold higher than A375 cells.

### 2.2. Combination of DOX and 43 °C Hyperthermia Decreases Cell Viability in A375 and MNT-1 Cell Lines

**Figure 2:**
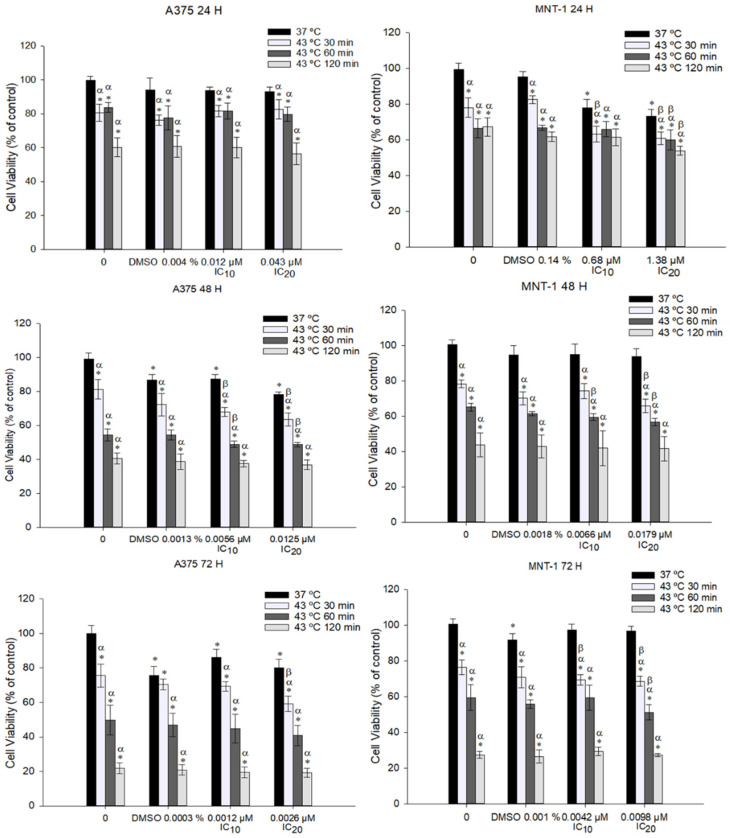
Effect of hyperthermia plus DOX on cell viability of A375 and MNT-1 cells. Cells were exposed to 43 °C for 30, 60, or 120 min, plus 0.012 μM or 0.043 μM and 0.68 μM or 1.38 μM during 24 h; 0.0056 μM or 0.0125 μM and 0.0066 μM or 0.0179 μM during 48 h; and 0.0012 μM or 0.0026 μM and 0.0042 μM or 0.0098 μM during 72 h; in cases of A375 or MNT-1, respectively. DMSO concentrations correspond to the equivalent percentage present in IC_20_ of each cell line and time exposure. DOX concentrations correspond to the calculated IC_10_ and IC_20_ for each time exposure and for each cell line. Cell viability was determined using MTT assay. Data are shown as mean ± standard deviation of two independent experiments with four technical replicates each. *—indicates statistical significance in comparison to the control 37 °C; α indicates statistical significance in comparison to the respective control of each condition at 37 °C; and β indicates statistical significance of the combined treatment in comparison to hyperthermia alone (*p* < 0.05).

To investigate if hyperthermia potentiates the effect of DOX, A375 and MNT-1 cell lines were exposed to 43 °C and to the calculated DOX IC_10_ and IC_20_ for 24, 48, and 72 h of exposure. Data showed that all tested time exposures to hyperthermia combined with the DOX IC_20_ significantly reduced MNT-1 viability after 24 h, when compared to hyperthermia alone or to DOX alone (Figure 2). However, A375 cells only showed similar results when exposed to 30 or 60 min hyperthermia and to DOX IC_10_ or IC_20_ after 48 h. In general, MNT-1 cell lines were shown to be more susceptible to the combined treatment than A375. Observing the obtained results, and preferring to use a low heating period and low DOX concentration with a significant potentiated effect, a heating period of 30 min at 43 °C and a 48-h exposure time to IC_20_ (0.0125 μM for A375 and 0.0179 μM for MNT-1) were selected for the following experiments.

### 2.3. Combination of DOX and 43 °C Hyperthermia Alters Cell Morphology

**Figure 3:**
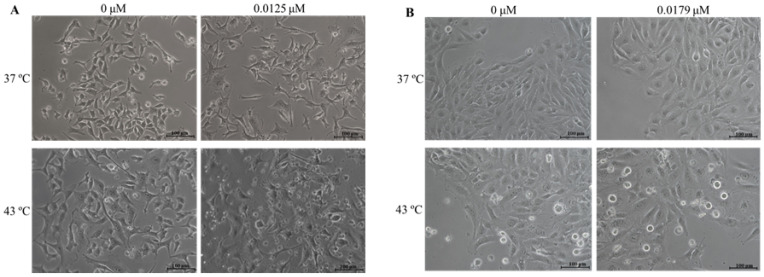
Effect of hyperthermia plus DOX on morphology of A375 and MNT-1 cells. Cells were exposed to 43 °C for 30 min and 0.0125 μM or 0.0179 μM of DOX, in case of A375 or MNT-1 cells, respectively. (**A**)—A375 cells; (**B**)—MNT-1 cells.

The effects of DOX and hyperthermia, both alone and in combined treatment, on A375 and MNT-1 cell morphologies were analyzed. Both cell lines were exposed to DOX at the concentration of IC_20_ for 48 h of exposure (0.0125 μM in case of A375 and 0.0179 μM in case of MNT-1), to 43 °C hyperthermia for 30 min, or to a combination of treatments. In the case of A375 cells, DOX alone caused cell stretching and flatness, as shown in Figure 3A. Hyperthermia alone had an identical effect, with the addition of roundness of some cells. The combined treatment increased the number of round cells, the number of vacuoles, and the number of cells in suspension. The effects of treatments in MNT-1 cell morphologies were more subtle, as represented in Figure 3B. In fact, DOX alone caused no relevant changes to cell morphology. However, some roundness and stretching were also observed with hyperthermia alone and combined with DOX, as well as more cells in suspension.

### 2.4. Combination of DOX and 43 °C Hyperthermia Induces Cell Cycle Arrest at G2/M Phase

**Figure 4:**
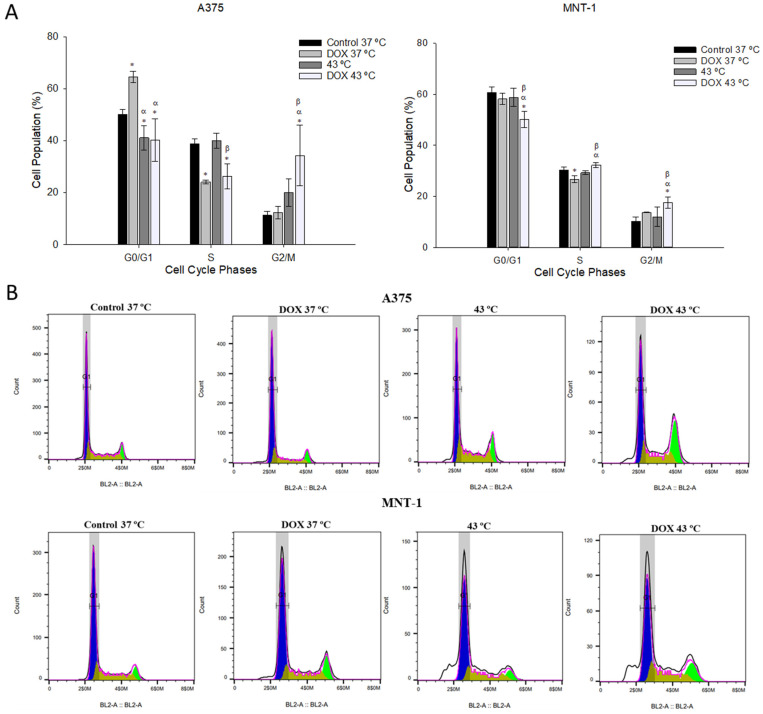
Effects of hyperthermia combined with DOX on cell cycle distribution. Cells were exposed to 43 °C for 30 min and 0.0125 μM or 0.0179 μM of DOX, in case of A375 or MNT-1 cells, respectively. (**A**) Cell cycle distribution (%) in A375 and MNT-1 cells; (**B**) histograms representative of cell distribution of A375 and MNT-1 cells. Data shown are mean values ± standard deviation of two independent experiments with two technical replicates each and each replicate with at least 5000 events. *—indicates statistical significance in comparison to the control 37 °C; α indicates statistical significance in comparison to the respective control of each condition at 37 °C; and β indicates statistical significance of the combined treatment in comparison to hyperthermia alone (*p* < 0.05).

In the attempt to analyze if an interference with the cell cycle progression was related to the chemotherapeutic effect of the DOX treatment combined with hyperthermia, both cell lines were exposed to 43 °C for 30 min and to the respective DOX IC_20_ for 48 h of exposure. Treatment with DOX alone significantly increased the percentage of A375 cells at the G0/G1 phase from 50% (control) to 66%, and subsequently decreased the number of cells at the S phase (from 40% to 24%), as can be seen in Figure 4. Contrarily, hyperthermia alone had no effect on A375 cell cycle dynamics. Nonetheless, hyperthermia and DOX combined caused a significant decrease in A375 cells at the G0/G1 and S phases and increased the percentage of A375 cells at the G2/M phase. In the case of MNT-1 cells, DOX alone significantly reduced the number of cells at the S phase. Hyperthermia alone had no impact on MNT-1 cell cycle dynamics, however, DOX plus hyperthermia significantly increased the percentage of MNT-1 cells at the G2/M phase by 7.5% and decreased the number of cells at the G0/G1 phase (a reduction of 10%).

### 2.5. Combination of DOX and 43 °C Hyperthermia Increases Intracellular ROS Levels

**Figure 5:**
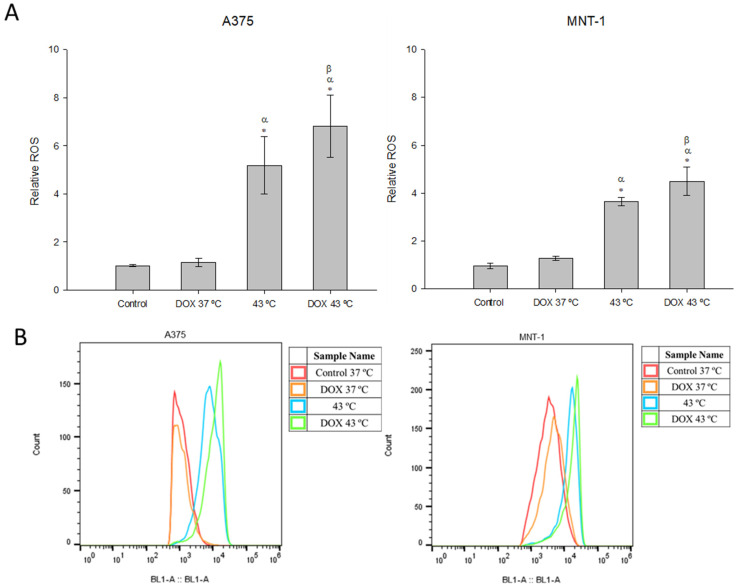
Effects of hyperthermia combined with DOX on production of intracellular ROS. Cells were exposed to 43 °C for 30 min and 0.0125 μM or 0.0179 μM of DOX for 48 h, in case of A375 or MNT-1 cells, respectively. (**A**) Relative abundance of intracellular ROS of A375 and MNT-1 cells; (**B**) histograms representative of abundance of intracellular ROS of A375 and MNT-1 cells. Data shown are mean values ± standard deviation of two independent experiments with two technical replicates each and each replicate with at least 5000 events. *—indicates statistical significance in comparison to the control 37 °C; α indicates statistical significance in comparison to the respective control of each condition at 37 °C; and β indicates statistical significance of the combined treatment in comparison to hyperthermia alone (*p* < 0.05).

The effects induced by the hyperthermia or DOX alone and in combination on the intracellular oxidative stress were evaluated by DCFH-DA probe and flow cytometry. Cells exposed to DOX alone showed no alterations on the production of ROS, despite a slight increase in MNT-1 cells (Figure 5). Hyperthermia alone induced a significant increase in ROS levels in A375 cells (5.1) and MNT-1 cells (3.6), compared to the control (defined as 1.0). Production of ROS was also significantly elevated by the combined treatment in both cell lines. In fact, the A375 cell line had a 1.3-times higher level of ROS compared to hyperthermia alone and a 6.8-times higher level compared to the control, while MNT-1 cell line had a 1.2-times higher level of ROS compared to hyperthermia alone and a 4.2-times higher level compared to the control.

### 2.6. Combination of DOX and 43 °C Hyperthermia Induces Apoptosis in MNT-1 Cells

**Figure 6:**
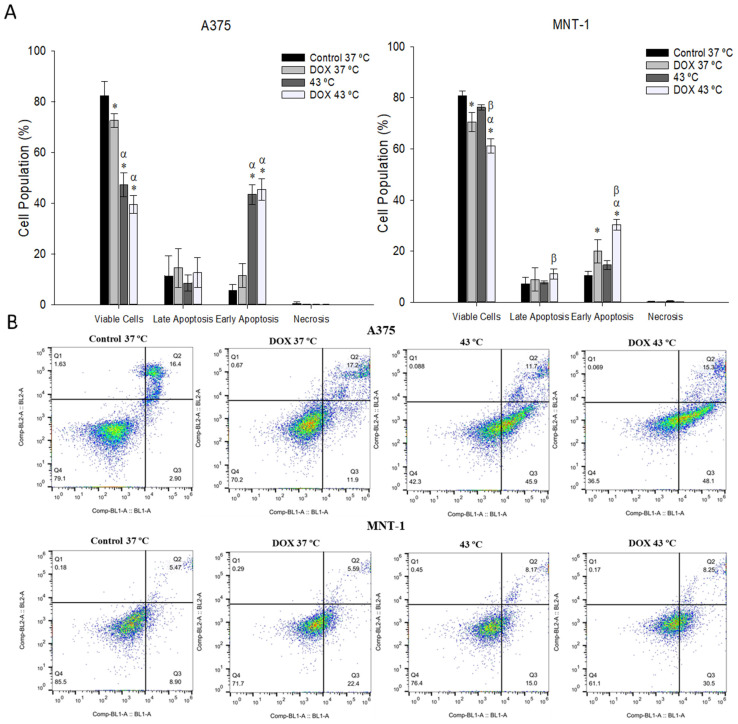
Effects of hyperthermia in combination with DOX on apoptotic profile. Both cell lines were exposed to 43 °C for 30 min and A375 cells were treated with 0.0125 μM and MNT-1 cells with 0.0179 μM of DOX for 48 h. (**A**) Percentage of apoptotic cells after treatment in populations corresponding to viable and non-apoptotic, early and late apoptotic A375 and MNT-1 cells; (**B**) histograms representative of Annexin V-FITC. Data shown are mean values ± standard deviation of two independent experiments with two technical replicates each and each replicate with at least 5000 events. *— indicates statistical significance in comparison to the control 37 °C; α indicates statistical significance in comparison to the respective control of each condition at 37 °C; and β indicates statistical significance of the combined treatment in comparison to hyperthermia alone (*p* < 0.05).

Apoptosis was also measured after both cell lines were exposed to 43 °C for 30 min and to the respective DOX IC_20_ for 48 h of exposure. As shown in Figure 6, in the case of the A375 cell line, there was an abrupt decrease in viable cells when treated with hyperthermia and with both treatments, caused by an increase in cells in early apoptosis when treated with hyperthermia (43%) and when treated with hyperthermia and DOX (46%), compared to the control (5.5%). MNT-1 cells had a decrease in viable cells when treated with DOX alone and with hyperthermia plus DOX. Additionally, the number of cells in late apoptosis increased when treated with both treatments, compared to hyperthermia alone. Both DOX alone and DOX plus hyperthermia increased the number of MNT-1 cells in early apoptosis (21% and 31%, respectively), compared to the control (11%).

## 3. Discussion

Melanoma is among the most aggressive and most difficult to treat types of cancer, causing over 9000 deaths each year [41]. DOX is a widely used anticancer drug but melanoma patients are not sensitive to this drug, with a response rate no higher than 15% [42]. With the application of hyperthermia simultaneously, it is possible to achieve higher DOX concentration in the tumor, increasing response rates. In the present study, the effects of combining hyperthermia and DOX on human A375 and MNT-1 melanoma cells towards the treatment of melanoma skin cancer were investigated.

The exposure of A375 and MNT-1 cells to DOX alone significantly decreased the cellular viability in a dose- and time-dependent manner. Additionally, MNT-1 cells were demonstrated to be less sensitive to DOX than A375 cells, having higher ICs for all the tested time exposures (Table 1). This can be correlated to the presence of melanin in MNT-1 cells, which was reported to bind to DOX and decrease drug activity [43]. Both DOX IC_10_ and IC_20_ corresponding to each time exposure and to each cell line were tested in combination with 43 °C. The A375 cell line only showed a potentiation when exposed to DOX for 48 h, while MNT-1 cells were more susceptible to the combined treatment. These results demonstrate that combining hyperthermia with DOX can be a better option for cells more resistant to DOX, such as pigmented cells. Other studies have already determined that the effects caused by DOX in melanoma cells are enhanced by hyperthermia [44,45,46]. Considering the obtained results from the combined treatment in the present work, an exposure time of 30 min to 43 °C and 48 h to DOX IC_20_ (0.0125 μM to A375 and 0.0179 μM to MNT-1) were selected for the succeeding experiments. It was observed that these conditions altered cell morphology, provoking flatness, stretching, and roundness. These effects were more perceptible in A375 cells.

Further experiments were realized to assess the potential mechanisms responsible for the cytotoxic effects of DOX combined with hyperthermia. Cell cycle is a cellular process common to all cells and important for cell proliferation [47]. To avoid errors, this process is controlled by specific molecular mechanisms that guarantee genome integrity [48]. In fact, when DNA damage is detected, these checkpoints cause cell cycle arrest, allowing DNA repair or causing cell death depending on the type of damage and in which cell cycle phase it is detected [49]. However, cancer cells have an unregulated cell cycle that causes uncontrolled cell proliferation [50]. Thus, cell cycle disruption is one of the main options for cancer treatment using agents that act in components of these pathways [51]. Flow cytometry was used to analyze A375 and MNT-1 cell cycles after treatments. Hyperthermia alone caused no significant effect in both cell lines. The treatment with DOX alone caused a significant arrest in A375 cells at the G0/G1 phase, resulting in a decrease of the percentage of cells at the S phase. In MNT-1 cells, DOX alone caused a slight but significant decrease of the percentage of cells at the S phase, but despite the higher concentration of drug used compared to the one used in the A375 cells, the treatment with DOX alone caused no cell cycle arrest in these cells. Contrary to our data, DOX alone was shown to cause an arrest at the G2/M phase in B16V and B16-F10 melanoma cells [52,53] and other types of cancer cells [54,55]. In our work, the combined treatment had a similar effect in both cell lines, causing an arrest at G2/M phase, compared to the control. However, A375 cells suffered a reduction of cells at both G0/G1 and S phases, while MNT-1 cells only showed a reduction of cells at the G0/G1 phase. These results showed that the combined treatment induced different effects from the treatments conducted alone, namely cell arrest and diminished cell proliferation, even when the separated treatments did not cause an effect. Few studies have focused on the effects of free DOX combined with hyperthermia on cell cycle dynamics, but none of which analyzed skin cancer cell lines. Wang et al. [56] reported that hyperthermia for 15 min or 30 min in combination with DOX (1 μM) caused a slight cell cycle arrest at the G2/M phase of HepG2 cells, similarly to our results, but the difference was not significant [56]. However, even though the concentration of DOX was higher than the ones used in our study, the temperature applied was 42 °C and some studies suggested that DOX requires temperatures above 42 °C to cause significant synergism [56,57]. Additionally, hyperthermia was performed pre-exposure to DOX, and the literature shows that better results are obtained when the drug is applied before or simultaneously with hyperthermia [57,58]. Besides, time exposure was only 24 h.

Oxidative stress and ROS production have been associated with certain types of human cancers, such as melanoma [59]. ROS can be produced in organisms by radiation, biotransformation of dietary chemicals, and some diet components [60], and are involved in each step of cancer development [61]. Several mechanisms can increase the intracellular ROS in cancer cells, such as the activation of oncogenes, high metabolism, mitochondrial dysfunction, and inactivation of tumor suppressor genes [61]. Despite this contribution to the development of tumors cells, enhanced intracellular ROS is considered a therapeutic target capable of inducing severe damage in cellular components, such as proteins, lipids, and chromosomes [62,63]. In fact, when cancer cells reach certain limits of ROS levels, the cells undergo apoptosis and are eliminated [60]. Actually, both hyperthermia and chemotherapeutic agents promote ROS production [64,65]. Indeed, many studies reported that DOX improved ROS production in diverse cell lines, and was considered the main cause of the cardiotoxicity observed when patients are exposed to an elevated amount of DOX, proving the need of using small concentrations of this drug [66,67,68,69]. Upon administration, DOX localizes mainly to the inner mitochondrial membrane, causing cellular toxicity through enhanced ROS production [70]. This overproduction takes place inside the mitochondria and is mediated by the mitochondrial NADPH oxidase (mitoNOX), as well as by cytochrome p450 and endothelial nitric oxide synthase [71,72]. Here, DOX alone, in the small concentrations used, was not able to significantly increase ROS production in both cell lines. On the contrary, hyperthermia alone significantly increased ROS production. However, the treatment with DOX plus hyperthermia was the one that caused the higher increase in ROS levels, significantly enhancing ROS production even when compared to hyperthermia alone. Wang et al. [56] also investigated the effects of hyperthermia plus DOX treatment in the ROS production of HepG2 cells and obtained similar results to ours, showing higher ROS levels than those with lone treatments. Moreover, we observed that the A375 cell line produced more ROS than the MNT-1 cell line when submitted to the treatments. This can be explained by the lack of melanin in A375 cells, since melanin has been reported as a protector against mitochondrial superoxide generation and mtDNA damage [73]. Since excessive levels of ROS may have a cytotoxic effect, causing the death of malignant cells, targeting these biochemical modifications might be a successful therapeutic strategy to prevent chemoresistance [74]. As melanomas are among the most drug-resistant cancers [75], and melanin has been suggested as a source of drug resistance [76], the present work shows that combining hyperthermia and DOX can help overcome these obstacles.

Apoptosis is characterized by diverse physiological modifications in cells, including the exposure of phosphatidylserine in cell surfaces, which can be recognized by its affinity for annexin V, a phospholipid-binding protein [77]. Therefore, the effects of hyperthermia combined with DOX on the induction of apoptosis were investigated. Previous studies have shown that hyperthermia (43 °C and 45 °C) induces apoptosis through the activation of caspase 3 in B16-F10 and A375 melanoma cell lines [78,79]. DOX was also shown to induce apoptosis in B16-F10 melanoma cells [80,81]. To our knowledge, this is the first study on the effects of hyperthermia combined with DOX on induced apoptosis in melanoma cells. Here, both A375 and MNT-1 cell lines showed a significant increase in early apoptotic cells after the combined treatment, compared to the control and to DOX alone. However, in the case of the A375 cells, this increase was not statistically different from the percentage of early apoptotic cells after treatment with hyperthermia alone. Contrary to this, the apoptotic profile of MNT-1 cells suffered no changes after treatment with hyperthermia alone. These results suggested that the induction of apoptosis in MNT-1 cells following the treatment with hyperthermia plus DOX could explain the reduced cell viability caused by the combined treatment.

There are clinical trials reporting that the treatment of local and disseminated malignant melanoma is improved not just by a combination of chemotherapeutic agents with regional hyperthermia [82,83,84], but also with whole-body hyperthermia in the case of more distant metastasis [85]. In the last few years, the development of nanoparticle formulations for therapeutic applications has been an important focus area due to their efficient delivery to cancer cells, avoiding their accumulation in healthy cells [86]. In fact, magnetic nanoparticles can help overcome the observed side effects of whole-body hyperthermia and positive results were already obtained in melanoma treatment [87,88,89]. Furthermore, DOX-loaded magnetic nanoparticle efficacy was already evaluated [90]. Thus, with the recent advantages, the transposition of our work to clinical practice is possible.

Our results reinforce the idea that DOX-combined-with-hyperthermia treatment might be an effective therapy against drug-resistant melanomas and elucidate some of the mechanisms involved. In fact, it was demonstrated that small concentrations of DOX that have little or no effects when used alone are potentiated by hyperthermia, causing cell cycle arrest and oxidative stress resulting in apoptosis. However, further studies should be performed to fully comprehend the involved mechanisms, such as analyzing alterations in the expression of genes involved in cell cycle control; the characterization of caspase 3, 8, and 9 activity; and in vivo studies.

## 4. Materials and Methods

### 4.1. Cell Lines and Cell Culture

Human melanoma cell line A375 was purchased from the European Collection of Authenticated Cell Cultures (ECACC) and supplied by Sigma-Aldrich (Madrid, Spain) and the MNT-1 melanoma cell line was kindly provided by Dr. Manuela Gaspar (iMed.ULisboa, Lisbon, Portugal). Cell lines were cultured in cell culture plastic flasks (SPL Life Sciences, Gyeonggi-do, Korea) in Dulbecco’s Modified Eagle Medium (DMEM; Gibco, Life Technologies, Grand Island, NY, USA), supplemented with 10% (*v*/*v*) fetal bovine serum (FBS; Gibco, Life Technologies, Grand Island, NY, USA), 2 mM L-glutamine, 1% pen/strep (100 U/mL penicillin, 100 μg/mL streptomycin; Grisp, Porto, Portugal), and 2.5 μg/mL fungizone (Gibco, Life Technologies, Grand Island, NY, USA), in a humidified incubator at 37 °C and 5% CO_2_. Cell confluence and morphology were monitored frequently, and subcultures were performed when monolayers reached 75–80% confluence.

### 4.2. Determination of Cell Viability

#### 4.2.1. Exposure to DOX

Initially, a stock solution was made by dissolving doxorubicin hydrochloride (≥98%; Cayman Chemical, USA) in dimethyl sulfoxide (DMSO, ≥99.5%; Sigma-Aldrich, St. Louis, MO, USA). Cell lines were seeded in 96-well plates and incubated at 37 °C with 5% CO_2_ for 24 h. Then, the cell culture medium was aspirated, and cells were incubated with a range of nine concentrations of DOX diluted in culture medium (0.001, 0.005, 0.01, 0.05, 0.1, 0.5, 1, 5, and 10 μM). The plates were incubated at 37 °C and 5% CO_2_ during 24, 48, and 72 h intervals, and thereafter viability was measured.

#### 4.2.2. Exposure to Hyperthermia Combined with DOX

Cells were seeded into 96-well plates and allowed to adhere for 24 h. Afterwards, medium was replaced by the DOX IC_10_ and IC_20_ for each cell line and time exposure (0.012 μM or 0.043 μM and 0.68 μM or 1.38 μM during 24 h; 0.0056 μM or 0.0125 μM and 0.0066 μM or 0.0179 μM during 48 h; and 0.0012 μM or 0.0026 μM and 0.0042 μM or 0.0098 μM during 72 h; in case of A375 or MNT-1, respectively). Then, the plates were exposed to 43 °C for 30, 60, or 120 min, or incubated at 37 °C. The plates exposed to 43 °C were transferred to the incubator at 37 °C after the hyperthermia treatment. Cell viability was measured after 24, 48, and 72 h post-exposure.

#### 4.2.3. Cell Viability Measurements

Determination of cell viability was based on the colorimetric MTT (3-(4,5-dimethyl-2-thiazolyl)-2,5-diphenyl-2H-tetrazolium bromide) assay (98%; Sigma-Aldrich, St. Louis, MO, USA). Upon cell exposure, 50 μL of MTT (1.0 mg/mL in phosphate-buffered saline) was added to each well. After 4 h incubation at 37 °C, the medium with MTT from each well was replaced with 150 μL of DMSO to dissolve the formazan crystals. The plates were shaken in the dark for 2 h. The optical density was measured at 570 nm using a microplate reader (Synergy HT^®^ Multi-Mode; BioTek^®^, Vinooski, VT, USA). At least two independent assays were performed with 4 replicates each. The cells without exposure were used as control. The cell viability was calculated using Equation (1).

(1) $$Cell\;Viability\;\left(\%\;of\;control\right)=\frac{\mathrm{Sample}\;\mathrm{Absorbance}\;-\;\mathrm{Blank}\;\mathrm{Absorbance}\;}{\mathrm{Control}\;\mathrm{Absorbance}\;-\;\mathrm{Blank}\;\mathrm{Absorbance}}\times 100\;$$

### 4.3. Cell Morphology

A375 and MNT-1 cells were seeded in 12-well plates and allowed to adhere, as described above. Thereafter, cells were exposed to DOX at 0.0125 or 0.0179 μM, equivalent to DOX IC_20_ of A375 and MNT-1, respectively, and to 43 °C hyperthermia for 30 min or incubated at 37 °C. After exposure to 43 °C, cells were incubated at 37 °C until 48 h incubations were completed. Then, cell morphology was analyzed and representative images were captured on an inverted phase-contrast Eclipse TS100 microscope (Nikon, Tokyo, Japan).

### 4.4. Cell Cycle Analysis

Cells were seeded in 12-well plates and after adhesion the medium was removed and replaced by DOX at 0.0125 μM or 0.0179 μM equivalent to DOX IC_20_ of A375 and MNT-1, respectively. Next, cells were exposed to 43 °C for 30 min and then were incubated at 37 °C for 48 h. After incubation, cells were harvested, washed in PBS, fixed with 85% cold ethanol, and kept at −20 °C until analysis. Then, samples were washed, resuspended with PBS, and filtered on a nylon mesh to the test tubes. Cells were incubated with 50 μL RNAse (Sigma-Aldrich, St. Louis, MO, USA) for 10 min and then with 50 μL of propidium iodide (PI, ≥94%; Sigma-Aldrich, St. Louis, MO, USA) for at least 20 min in the dark and at room temperature. Propidium iodide-stained cells were analyzed on an Attune^®^ Acoustic Focusing Cytometer (Applied Biosystems, Termo Fischer Scientific, Agawam, MA, USA) and the percentages of cells at the G0/G1, S and G2/M phases were determined using the FlowJo software (FlowJo LLC, Ashland, OR, USA). Two independent assays with two replicates each were performed for each treatment, and for each sample at least 5000 events were acquired.

### 4.5. Analysis of Intracellular ROS

Intracellular ROS accumulation was determined using the probe 2′,7′-dichlorofluorescein diacetate (DCFH-DA; Sigma-Aldrich, St. Louis, MO, USA), which in the presence of ROS is converted to the fluorescent compound 2′,7′–dichlorofluorescein (DCF). Briefly, cells were seeded in 12-well plates and incubated at 37 °C with 5% CO_2_ for 24 h for adhesion. Then, medium was replaced by fresh medium with DOX at the concentrations of 0.0125 μM or 0.0179 μM, respectively, for A375 and MNT-1. Plates were exposed to 43 °C for 30 min and then incubated at 37 °C for 48 h. Then, cells were washed with PBS and incubated for 30 min with 10 μM DCFH-DA in culture medium with 2% FBS. After staining, cells were detached and DCF fluorescence was analyzed within 45 min on an Attune^®^ Acoustic Focusing Cytometer (Applied Biosystems, Termo Fischer Scientific, MA, USA).

### 4.6. Cell Apoptosis Assay

To measure the ratio of the apoptosis, an Annexin V-FITC Apoptosis Detection kit (BD Biosciences, Franklin Lakes, NJ, USA) was used. A375 and MNT-1 cells were allowed to adhere in 6-well plates. After treatment of 30 min at 43 °C and 0.0125 μM or 0.0179 μM of DOX, corresponding to DOX IC_20_ for 48 h, for A375 or MNT-1, respectively, cells were collected, counted, and then washed with PBS twice after centrifugation (300× *g*, 5 min, 4 °C). Then, cells were resuspended in binding buffer to a concentration of 1 × 10^6^ cells/mL, and 5 μL of Annexin V-FITC and 5 μL of PI were added to 100 μL of cell suspension. Samples were incubated in the dark for 15 min and 400 µL of binding buffer was added to each sample. Analysis was performed in the next hour on an Attune^®^ Acoustic Focusing Cytometer (Applied Biosystems, Termo Fischer Scientific, MA, USA) and data acquired with FlowJo (FlowJo LLC, Ashland, OR, USA).

### 4.7. Statistical Analysis

Data are expressed as mean ± standard deviation. Statistical analysis was performed using SigmaPlot version 14.0 (Systat Software, San Jose, CA, USA) for Windows. Data were analyzed by one-way ANOVA (*p* < 0.05) followed by Dunnett’s test (*p* < 0.05) in the case of the initial experiment with exposure to only DOX, and followed by Tukey’s test (*p* < 0.05) for multiple comparisons in the following experiments.

## Figures and Tables

**Table 1:** Inhibitory concentrations (ICs) obtained for 24, 48, and 72 h DOX exposure. Values are expressed in μM.

Cell Line	IC	24 H	48 H	72 H
A375	IC_10_	0.012	0.0056	0.0012
	IC_20_	0.043	0.0125	0.0026
	IC_50_	0.45	0.052	0.0111
MNT-1	IC_10_	0.68	0.0066	0.0042
	IC_20_	1.38	0.0179	0.0092
	IC_50_	4.88	0.102	0.0391

## Data Availability

All data reported in this paper are contained within the manuscript.
